# Mandibular fractures: a comparative analysis between young and adult patients in the southeast region of Turkey

**DOI:** 10.1590/S1678-77572010000100005

**Published:** 2010

**Authors:** Serhat ATILGAN, Behçet EROL, Ferhan YAMAN, Nezih YILMAZ, Musa Can UCAN

**Affiliations:** 1 DDS, PhD, Assistant Professor, Department of Oral and Maxillofacial Surgery, Faculty of Dentistry, Dicle University, Diyarbakir - Turkey.; 2 DDS, PhD, Professor, Department of Oral and Maxillofacial Surgery, Faculty of Dentistry, Dicle University, Diyarbakir - Turkey.; 3 DDS, PhD, Research Assistant, Department of Oral and Maxillofacial Surgery, Faculty of Dentistry, Dicle University, Diyarbakir - Turkey.; 4 DDS, Research Assistant, Department of Oral and Maxillofacial Surgery, Faculty of Dentistry, Dicle University, Diyarbakir - Turkey.

**Keywords:** Mandibular fracture, Young and adult patients, Retrospective study

## Abstract

**Objective:**

The purpose of this study was to review and compare the differences between mandibular fractures in young and adult patients.

**Material and Methods:**

Patients treated at the Oral and Maxillofacial Department of Dicle University during a five-year period between 2000 and 2005 were retrospectively evaluated with respect to age groups, gender, etiology, localization and type of fractures, treatment methods and complications.

**Result:**

532 patients were included in the study, 370 (70%) males and 162 (30%) females, with a total of 744 mandibular fractures. The mean age of young patients was 10, with a male-female ratio of 2:1. The mean age of adult patients was 28, with a male-female ratio of 3:1. The most common causes of injury were falls (65%) in young patients and traffic accidents (38%) in adults. The most common fracture sites were the symphysis (35%) and condyle (36%) in young patients, and the symphysis in adults (36%). Mandibular fractures were generally treated by arch bar and maxillomandibular fixation in both young (67%) and adult (39%) patients, and 43% of the adult patients were treated by open reduction and internal fixation.

**Conclusion:**

There was a similar gender, monthly and type of treatment distribution in both young and adult patients in the southeast region of Turkey. However, there were differences regarding age, etiology and fracture site. These findings between young and adult patients are broadly similar to those from other studies. Analysis of small differences may be an important factor in assessing educational and socioeconomic environments.

## INTRODUCTION

The facial area is one of the most frequently injured parts of the body^1-3^, and the mandible is one of the most commonly fractured maxillofacial bones^1,4,5^. Injuries of the maxillofacial area can be psychologically disturbing for patients and have a functional impact^6^.

Local patterns and causes of mandible fractures vary considerably among different study populations, and recent overall shifts in the mechanism of injury and age distribution of patients sustaining such injuries are well documented^7-10^. There is an emerging trend towards an increase in the frequency of violent mechanisms of fracture and in the proportion of adolescents and young adults sustaining such injuries. These trends seem to hold true in urban settings in particular^11-13^.

Epidemiological studies regarding maxillofacial fractures are helpful in evaluating the quality of patient care and in planning preventive strategies. These studies are also valuable in identifying new frequencies and patterns of these fractures^6^.

Limited information is available regarding mandibular fracture patterns in Turkey, and no comparative studies have been undertaken in the southeast region of the country. The aim of this study was to compare the etiology and frequency of mandibular fractures in young and adult patients in southeast Turkey.

## MATERIAL AND METHODS

This was a retrospective study of all mandibular fractures seen at the Oral and Maxillofacial Surgery Department of Dicle University. During the 5-year period from 2000 to 2005, data (clinical records, patients' files) were reviewed and analyzed in terms of age, gender, etiology, anatomical site of fracture, monthly distribution, treatment methods and complications. Patients were divided into two subgroups: ‘young' patients consisting of children (0-12 years old) and adolescents (12-18 years old), and ‘adults' (> 18 years old). Fracture sites were assigned to one of seven different mandibular subsites; including the symphysis/ parasymphysis, body, angle, ramus, condyle and alveolus. In addition, the cause of injury was also divided into 7 categories: road traffic, falls, interpersonal violence, kicks from animals, gunshots, sports accidents and others. Percentages and means were calculated using Microsoft Excel software.

## RESULTS

### Age and gender distribution

During the 5-year study period (2000-2005) 532 patients sustained 744 mandibular fractures. Their ages ranged from 1 to 80 with a mean age of 21. Of these 532 patients, 370 (70%) were male and 162 (30%) female (ratio: 2.2:1). The number of young patients was 302, with 422 fractures, and the number of adults was 230, with 322 fractures (Table 1).

**Table 1:** Gender distribution of all patients with mandibular fractures

	**Young (%)**	**Adult (%)**	**Total (%)**
Male	191 (63)	179 (78)	370 (70)
Female	111 (37)	51 (22)	162 (30)
Total	302 (100)	230 (100)	532 (100)

The age of the young patients ranged from 1 to 18 with a mean age of 10. There were 214 (71%) children and 85 (29%) adolescents. The majority of young patients (46%) were between the ages of 6 and 12. The other groups' levels were broadly similar (0-5 years: 27%, 13-18 years: 29%). Of the young patients, 111 were female (37%) and 191 male (63%) (Table 1).

The ages of the adult patients ranged from 19 to 80, with a mean of 28. Most adult patients were in the 19-29 age group (130 patients, 55%). The majority of patients were male (n=179, 78%) and 51 patients were females (22%) (Table 1).

### Etiology

Different causes were involved in young and adult patients (Table 2). The most common cause of injury in young patients was falls (65%), while road traffic accidents predominated in adult patients (88%).

**Table 2:** Etiology of mandibular fractures in all patients

**Type**	**Young (%)**	**Adult (%)**	**Total (%)**
Road Traffic	65 (22)	88 (38)	153 (28)
Falls	195 (65)	53 (23)	248 (46)
Interpersonal violence	21 (7)	51 (22)	72 (13.5)
Animal kicks	10 (3.3)	12 (5.7)	32 (6.0)
Gunshots	2 (0.7)	17 (7.3)	19 (3.5)
Sports accidents	6 (1.8)	4 (1.7)	10 (1.8)
Others	1 (0.2)	5 (2.3)	6 (1.2)
Total	300 (100)	230 (100)	530 (100)

### Location of Fractures

The locations of mandibular fractures in young and adult patients are listed in Table 3, the most common fracture sites being the symphysis/ parasymphysis for all patients. For young patients the most common fracture site was the condyle (36%), followed by the symphysis/ parasymphysis (35%). The most frequent site in adults was the symphysis/parasymphysis (36%), followed by the condyle (20%) and body (20%).

**Table 3:** Site distribution of mandibular fractures in all patients

**Fracture site**	**Young (%)**	**Adult (%)**	**Total (%)**
Symphysis and parasymphysis	151 (35)	116 (36)	267 (36)
Body	31 (8) 64	(20) 95	(12)
Angle	40 (10) 60	(19) 100	(13)
Ramus	-	3 (1)	3(0.5)
Condyle	152 (36)	66 (20)	218 (30)
Alveolar	48 (11)	13 (4)	61(8.5)
Total	422 (100)	322 (100)	744 (100)

### Monthly Distribution

**Figure 1:**
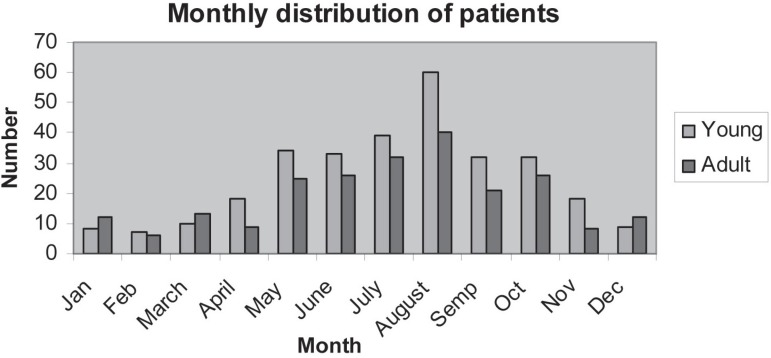
Monthly distribution

The monthly distributions in young and adult patients were broadly similar. The monthly distribution showed August to have the highest incidence, followed closely by July. The lowest incidence was observed during the winter months (Figure 1).

### Fracture Type

The most common fracture types were isolated fractures (56%) in young patients and multiple fractures (55%) in the adult patients (Table 5).

**Table 5:** Fracture type

**Fracture type**	**Number of young patients (%)**	**Number of adult patients (%)**	**Total (%)**
Isolated fractures	155 (56)	120 (44)	275 (100)
Multiple fractures	117 (45)	140 (55)	257* (100)
Total			532 (100)

* : 257 patients with 469 fracture lines

### Treatment of mandibular fractures

Different types of treatment were administered for mandibular fractures (Table 4). The majority of young patients (67%) were treated using the arch bar and maxillomandibular fixation (MMF). The most common method of treatment for adult patients was open reduction and internal fixation with miniplates (43%), followed closely by arch bar and MMF (39%).

**Table-4:** Relationship between fracture type and treatment methods

**Type of Treatment**	**Treatment Methods**	**Isolated fractures (young)**	**Multiple fracture (young)**	**Isolated fracture (adult)**	**Multiple fracture (adult)**
Observation (non-treated)	Recommendations (soft diet and oral hygiene)	6	0	6	0
					
Conservative treatment	a) Arch bar MMF (maxillomandibular fixation)	176	26	80	10
Open reduction	b) Circummandibular wires with an occlusal splint	2	15	1	6
	c) Inferior arch bar	12	0	2	0
	d) Interdental cerclage	19	0	3	0
	e) IVY Loops	18	0	6	0
					
	a) MPO(mini plate osteosynthesis)	0	24	7	92
	b) Reconstruction plate + graft	0	2	0	17

### Fracture type and treatment methods

Isolated mandibular fractures of the young patients were commonly treated by MMF (75.5%), followed by interdental cerclage (8.1%), ivy loops (7.7%), inferior arch bar (5.1%). Multiple fractures of the young patients were treated by mini plate osteosynthesis (MPO) (35%), MMF (35%), circummandibular wire with an occlusal splint (22%).

Among the adult patients, the most common treatment method was MMF for the isolated fracture. And also most MPO (73%) was the most common treatment method of the multiple fractures.

#### Complications

Complications were observes in twenty five patient (18 adult, 7 young patients). Soft tissue infection (5 young patients and 7 adult patients), osteomyelitis (1 young patient), pseudarthrosis (2 adult patients), delayed union (3 adult patients), anesthesia (1 young and 2 adult patients), temporomandibular joint disorders (4 adult patients) were detected in the follow up period. Proper treatments were performed in these cases.

### DISCUSSION

Fractures can occur at any age^26^ and the facial area is one of the most frequently injured parts of the body^10,14,22^. There is a lack of epidemiological comparative studies among young and adult patients.

In the literature, the frequency of facial fractures is lower in the young population than in the adult population^12,15^. However, the data on which this premise is based may be subject to alternative interpretations, and the true incidence of facial fractures in this region, especially in the young population, is much higher than previously reported. The reasons cited for this high incidence include the greater size of the young population, socioeconomic problems, and parents' careless attitudes.

In this study, young and adult males accounted for 69.5% of all patients with mandibular fractures, a level similar to those reported by Qudah, et al. ^25^, Dongas, et al. ^9^, Bremerich, et al. ^5^ and Edwards, et al.^10^ Both young and adult females are less affected than males, with an incidence of 30.5%. The findings from this study are consistent with those from previous research.

The highest incidence of mandibular fractures occurred in young patients aged 6-12 years, both male and female. The highest incidence of mandibular fractures in adult patients was observed in the 19-29 age group.

The main etiological patterns were different in young and adult patients. Our study was in agreement with other studies^5,24,30^ that falls were the most common cause of maxillofacial injuries in young patients, the second most common cause being road traffic accidents. However, studies from other parts of the world have reported that road traffic accidents were the leading cause of facial fractures in young adult patients^16,28^.

Among adult patients the main cause of mandibular fractures was traffic accidents, at a level of 3:1, followed by falls (23%) and interpersonal violence (22%).

These etiological pattern changes from region to region may be due to socio-economic problems, alcohol consumption, inadequate traffic laws, the stresses of residing in large cities etc. Some studies have determined physical assaults to be the predominant cause of mandibular fractures, followed by traffic accidents^2,9,10,11^. Additionally, other studies have reported that traffic accidents were the most common cause of mandibular fractures, as in our study^9,20^.

The most common site of mandibular fractures in adult patients was the symphysis and parasymphysis, followed by the condyle, body and angle. However, the mandibular symphysis/ parasymphysis and condyle were determined to be most common sites in young patients. These findings conflict with studies by Oji^24^ and Abiose^1^ in Ibadan, Nigeria, and by Ferreira^12^ in Portugal, in which the mandibular body was identified as the most common fracture site in adult patients. Our findings regarding young patients are consistent with those from previous studies^17,24^.

The anatomic location of fractures correlates significantly with the mechanism of patient injury, and knowledge of these associations should guide treating physicians in their diagnostic work-up of all head and neck trauma patients^19^. Victims of falls are significantly more likely to suffer parasymphyseal and condyle fractures but fewer body and angle fractures than might be expected. Automobile accident victims will more commonly have symphyseal/parasymphyseal fractures and fewer body fractures than expected^19^.

More fractures occurred in August and July, the holiday season. August and July also represent the middle of summer in Turkey, when outdoor activities and festivities are attended by large crowds. In addition, especially in this region, people sleep on roofs in the summer, which impacts on the level of falls.

The oral and maxillofacial surgeon now has many options for treating mandibular fractures. Nevertheless, complication rates are significant. Although some techniques may be better than others, no one technique can be used in all situations. In most cases, more than one comparable option is available. The patient and fracture should be properly evaluated, and the best options selected. Risks and benefits of each are then presented to the patient. In most situations both maxillomandibular fixation and rigid internal fixation are available to the patient. Successful implementation involves a thorough understanding of a technique and its limitations as well as the fixation requirements of the fracture. Only then can fractures be successfully treated and complications minimized^28,29,30^.

A conservative approach should be considered first for mandible fractures in young and adult patients. Many pediatric fractures are nondisplaced or green stick type fractures, and observation alone is adequate^15,18,21,29^. A soft diet is necessary for these patients, and displaced fractures in children and adults are treated using arch bar and MMF. The clinical outcome using a conservative approach is very successful. The fractures heal quickly and young patients are able to recover the function well. Unstable fractures can be secured with open reduction techniques and internal fixation^15,29^.

### CONCLUSION

There was a similar gender, monthly and type of treatment distribution among both young and adult patients in the southeast region of Turkey. However, there were differences regarding age, etiology, and fracture site. These findings between young and adult patients are broadly similar to those from other studies. Analysis of small differences may be an important factor in assessing educational and socioeconomic environments.
